# Two Decades of Publications in Journals Dedicated to Autoimmunity: A Bibliometric Analysis of the Autoimmunity Field from 2004 to 2023

**DOI:** 10.1007/s10238-024-01369-1

**Published:** 2024-06-04

**Authors:** Sha-Sha Tao, Jian Tang, Xiao-Ke Yang, Xi Fang, Qing-Qing Luo, Yi-Qing Xu, Man Ge, Fan Ye, Peng Wang, Hai-Feng Pan

**Affiliations:** 1 Department of Epidemiology and Biostatistics, School of Public Health, Inflammation and Immune Mediated Diseases Laboratory of Anhui Province, Anhui Medical University, Hefei, 230032 Anhui China; 2 Preventive Medicine Experimental Teaching Center, School of Public Health, Anhui Medical University, Hefei, 230032 Anhui China; 3 Department of Rheumatology and Immunology, The First Affiliated Hospital of Anhui Medical University, Hefei, 230032 Anhui China; 4 Department of Environmental Health, School of Public Health, Shanxi Medical University, TaiYuan, 030001 Shanxi People’s Republic of China; 5 Teaching Center for Preventive Medicine,School of Public Health, Anhui Medical University, Hefei, 230032 Anhui China

**Keywords:** Autoimmunity, Bibliometric analysis, Journals dedicated to autoimmunity, Citespace, VOSviewer

## Abstract

To carry out an in-depth analysis of the scientific research on autoimmunity, we performed the first bibliometric analysis focusing on publications in journals dedicated to autoimmunity (JDTA) indexed by science citation index during the period 2004–2023. Using bibliometric analysis, we quantitatively and qualitatively analyzed the country, institution, author, reference and keywords information of publications in JDTA, so as to understand the quantity, publication pattern and publication characteristics of these publications. The co-occurrence networks, clustering map and timeline map were created by CiteSpace and VOSviewer software to visualize the results. The CiteSpace was also used to analyze the strongest citation burst of keywords, which could describe the frequency, intensity and time period of high-frequency keywords, and indicate the research hotspots in the field. A total of 5 710 publications were analyzed, and their annual distribution number was basically stable from 2004 to 2023, fluctuating around 300. The United States and Italy led the way in terms of the number of publications, followed by France and China. For international cooperation, the developed countries represented by the United States cooperate more closely, but the cooperation was localized, reflecting that there was no unified model of autoimmunity among countries. UDICE-French Research Universities had the greatest number of publications. Subsequently, the number of publications decreased slowly with the ranking, and the gradient was not large. Eric Gershwin and Yehuda Shoenfeld stood out among the authors. They had an excellent academic reputation and great influence in the field of autoimmunity. The results of keyword analysis showed that JDTA publications mainly studied a variety of autoimmune diseases, especially SLE and RA. At the same time, JDTA publications also paid special attention to the research of cell function, autoantibody expression, animal experiments, disease activity, pathogenesis and treatment. This study is the first to analyze the publications in JDTA from multiple indicators by bibliometrics, thus providing new insights into the research hotspots and development trends in the field of autoimmunity.

## Introduction

With the passage of time and the continuous development of disciplines, there has been a considerable increase in scientific publications (including in the field of autoimmunity). When researchers look up publications in a certain field, a large number of literature are available to read and study. However, they are also faced with many problems. First, the existing literature has a wide range of topics, different research types, different research depths, and uneven quality. Secondly, the publication patterns of different journals are flourishing, and the types and requirements of publications are also different, especially the publications on autoimmunity topics (POAT). POAT has been published in a variety of journal types, including journals dedicated to autoimmunity (JDTA), immunology subspecialty journals, and general medical journals. Among them, there are three main JDTA indexed by science citation index (SCI), including Autoimmunity Reviews [1], Journal of Autoimmunity [2] and Autoimmunity [3].

These three journals cover all types and different levels of articles in the field of autoimmunity due to their large impact factor span (from 3.5 to 13.6) and comprehensive article types, which is of great significance for guiding the research in the field of autoimmunity. A large number of high-quality autoimmunity-related articles (ARAs) were published in these three journals, which closely follow the research hotspots and are in the forefront of the field, and have a good guiding role for researchers who want to carry out some deep and innovative research [4–8]. At the same time, they also published some basic ARAs, which is very helpful for novice researchers to quickly and systematically understand and learn professional knowledge in this field [9–11]. Therefore, analyzing the characteristics of the articles published in these three journals will help researchers better understand the basis, frontier, hot spots and challenges in the field of autoimmunity and is also helpful for readers to quickly obtain an overview of the development data of these journals, such as content, subject, and influencing factors.

Bibliometric analysis is a kind of literature analysis method that can quantitatively and qualitatively analyze the quantity, publication pattern and publication characteristics of published literature in a particular field [12]. It can analyze the author, keywords, periodicals, state, institution references and other information of publications, using visualization tools, including CiteSpace, VOSviewer, and HistCite tools, so as to provide clues and theoretical basis for studying the research trends and focus of various disciplines [13–15].

Therefore, the objective of this study was to analyze the characteristics of articles published in these three main JDTAs using bibliometric methods, which will help researchers to better understand the fundamentals, frontiers, hotspots and challenges in the field of autoimmunity.

## Methods

### Journal Selection

Web of Science (WOS), one of the world’s most trusted publishers of global citation databases, is a leading platform for scientific research [16]. The JDTAs were identified by searching the WOS Core Collection (WOSCC) database using “autoimmunity” as the search term in the “Publication/Source Title” field. Finally, three leading journals were selected, including “Autoimmunity Reviews” [1], “Journal of Autoimmunity” [2] and “Autoimmunity” [3].

“Autoimmunity Reviews” usually publishes cutting-edge structured reviews authored by leading experts in the field, covering various topics in autoimmunity. The articles will encompass all areas of autoimmune research, with a focus on bridging the gap between basic and clinical sciences.

The “Journal of Autoimmunity” publishes papers relating to all aspects of autoimmunity, including mechanisms of self-recognition, regulation of autoimmune responses, experimental autoimmune diseases, diagnostic autoantibody testing, epidemiology, pathophysiology, and therapy. It particularly emphasizes but is not limited to papers involving genetics, molecular biology, and cell biology in the discipline.

“Autoimmunity” is an internationally peer-reviewed journal that publishes articles on cellular and molecular immunology, immunogenetics, and molecular biology related to immunity and autoimmunity. The journal focuses on immune system cells, with a particular interest in the regulatory mechanisms of T cells and B cell antibody responses, emphasizing the intrinsic epigenetic and metabolic processes within immune cells.

### Data Processing

**Fig. 1:**
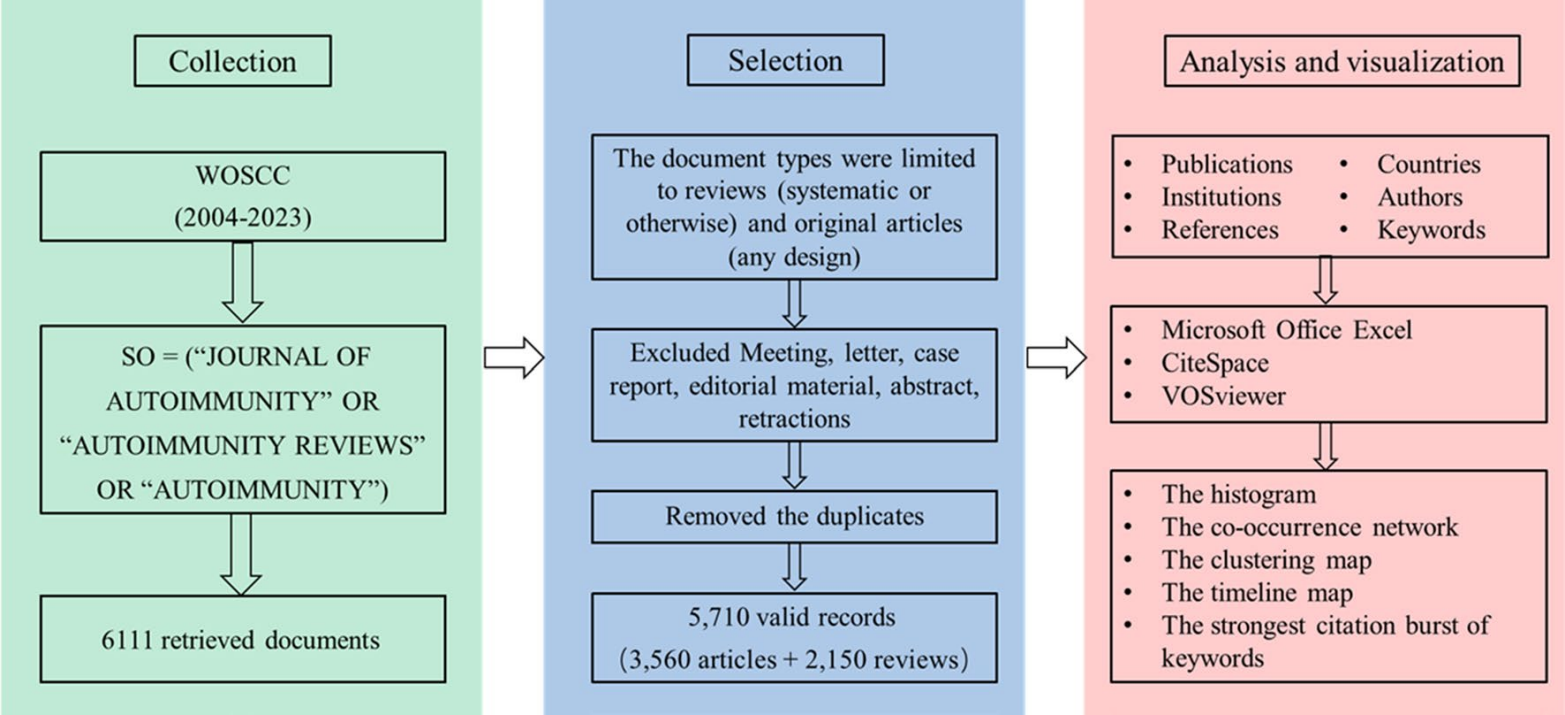
Flowchart of literature selection

Literature published in these three JDTAs was searched in WOSCC, and the detailed query formulation was described as follows: SO = (“JOURNAL OF AUTOIMMUNITY” OR “AUTOIMMUNITY REVIEWS” OR “AUTOIMMUNITY”). Because WOSCC only covers the literature published in these three JDTAs since 2004, the literature we analyzed was from January 1, 2004 to June 21, 2023. The document types were limited to reviews (systematic or otherwise) and original articles (any design). Meeting, letter, case report, editorial material, abstract, retractions were excluded. The bibliometrics description of all documents, including types of documents, most prolific articles, authors, institutions, countries and so on was collected. A hierarchical filtering process was used to ensure all study types that were mutually exclusive and that each article was only indexed for 1 study type. We reviewed a random 100 sample of the records to ensure classification accuracy for specialty and study type. After removing the duplicates manually, a total of 5,710 publications, including 3,560 articles and 2,150 reviews, were selected as the final data set for further bibliometric analysis. The search and analysis processes are shown in Fig. 1. In our study, ethical review was not required.

### Statistical Analysis

The bibliometrics description of all documents was analyzed using the Microsoft Office Excel 2022, CiteSpace (v.6.2.R4 Advanced) [17] and VOSviewer (v.1.6.19) [18]. The histogram produced by Microsoft Office Excel showed the different number publications by different years, countries, institutions and authors. CiteSpace and VOSviewer were used to create co-occurrence networks, in which the size and color of the circles represent the number and year of publications, respectively, and the number and thickness of the lines between the circles represent the frequency and intensity of cooperation between the research objects, respectively [19]. The clustering map and timeline map were drawn by CiteSpace according to the logarithmic likelihood ratio of keywords and were used to describe the research direction and the change progress of research hotspots over time. When the clustering modular value Q (Modularity Q) > 0.3, the clustering contour index S (Mean Sihouette) > 0.5 indicates a reasonable clustering, with clear structure and high reliability. The clustering map and timeline map in this study all met the requirements. The CiteSpace software was used to analyze the strongest citation burst of keywords, which could describe the frequency, intensity and time period of high-frequency keywords, and indicate the research hotspots in the field [20, 21].

## Results

### Publication Numbers Sorted by Year

**Fig. 2:**
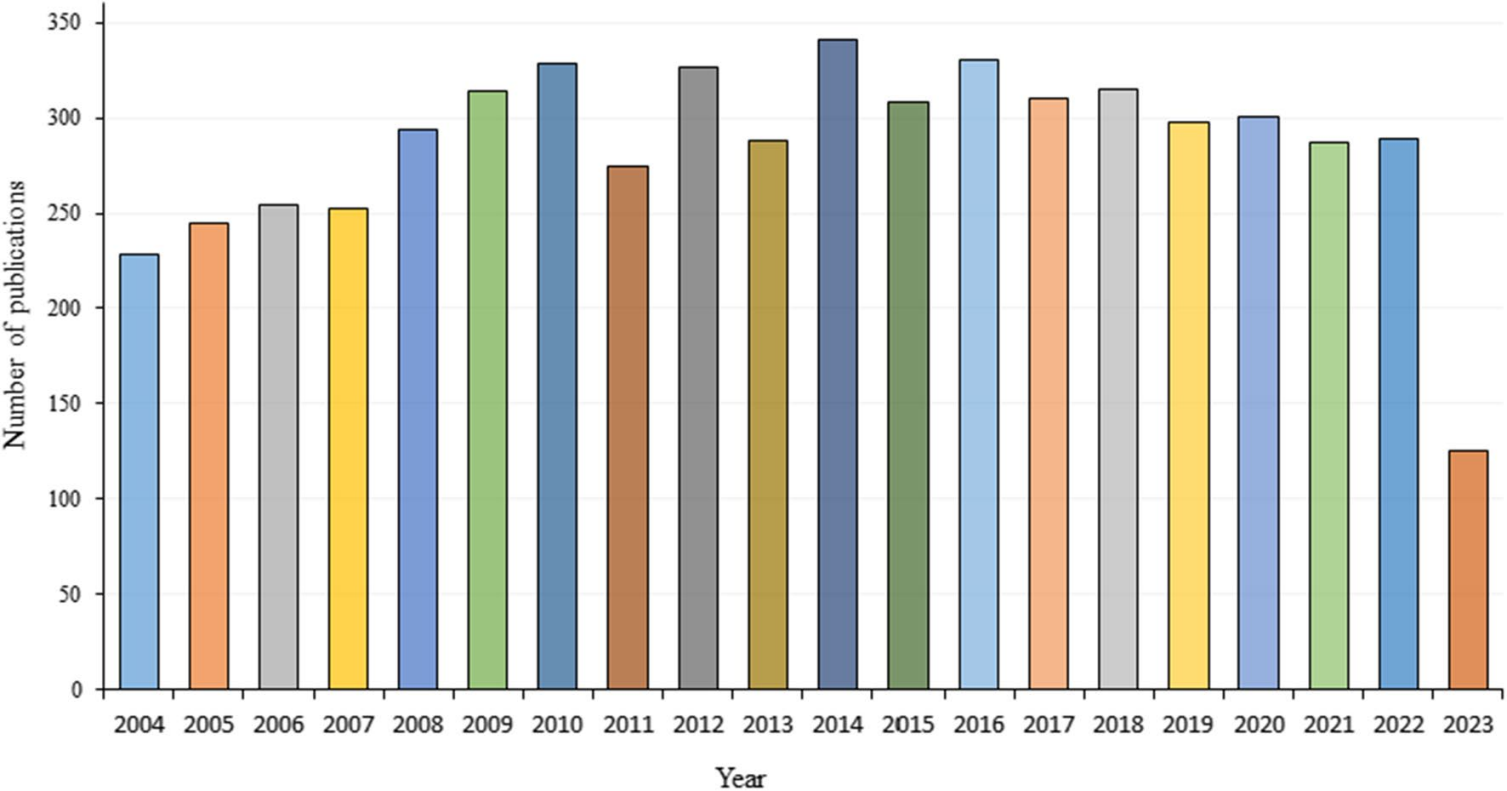
Annual distribution of publications from 2004–2023

A total of 5,710 articles were analyzed, of which 3,560 (62.35%) were original articles and 2,150 (37.65%) were reviews. From 2004 to 2010, the number of literature increased slowly and steadily, up to 329 papers, suggesting that more and more attention had been paid to the field of autoimmunity. From 2011 to 2017, the number of publications showed a slight fluctuation, with the annual number of publications ranging from 275 to 341. Since 2017, the annual number of publications stabled at about 300 (Fig. 2).

### Country Distribution of Publications

#### Country Co-occurrence Network

**Fig. 3:**
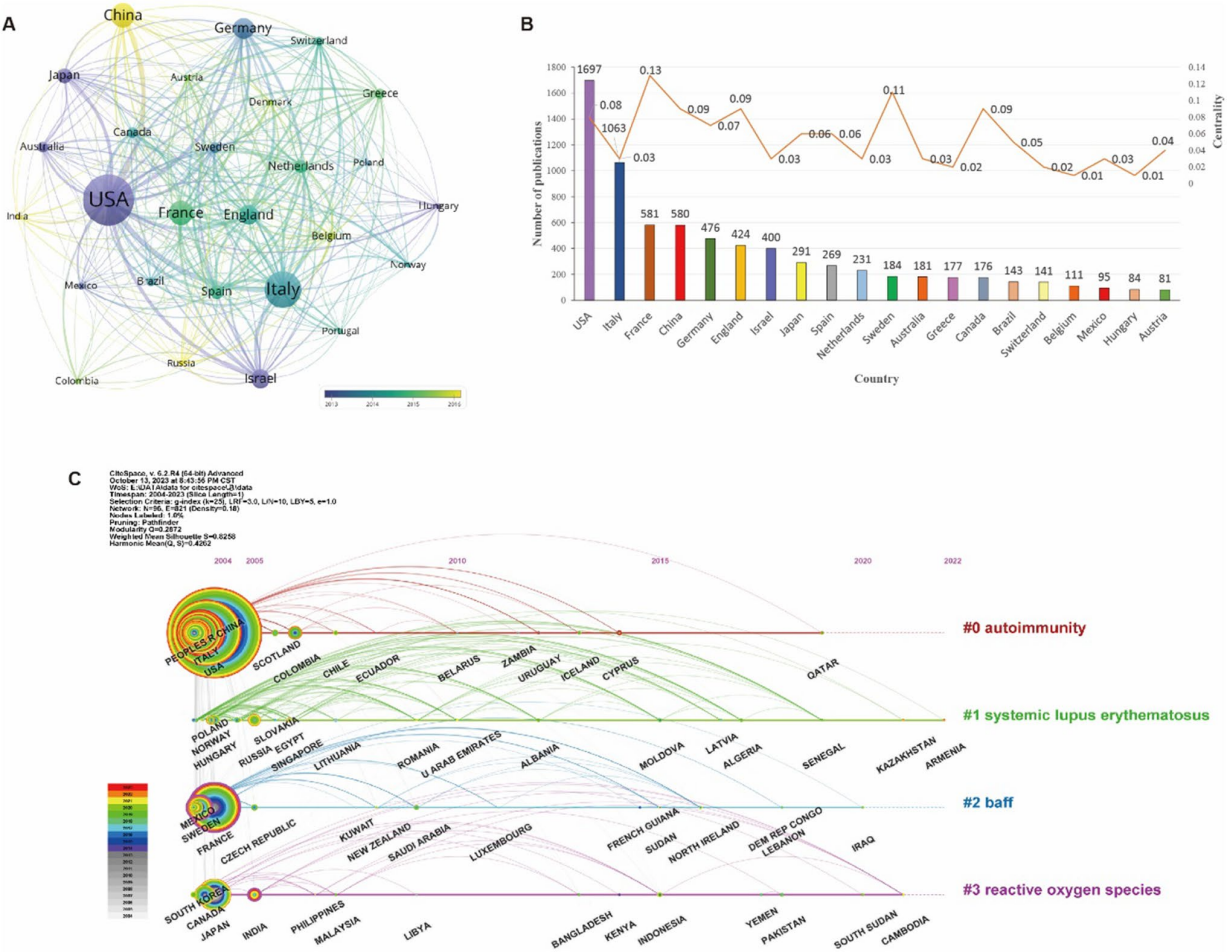
Analysis of the country distribution of publications. *Note* A: the co-occurrence network of countries; B: top 20 countries by number of publications and their centrality; C: the timeline map of countries

As shown in Fig. 3, among the 96 countries that have published papers in JDTA, the United States had the highest number of publications. The United States led the way with 1,697 (19.87%) publications, followed by Italy with 1,063 (12.44%), France with 581 (6.80%), China with 580 (6.80%), Germany with 476 (5.58%) and England with 424 (5.00%), and the remaining countries/regions had published less than 5.00% articles. In addition, France and Sweden played a “bridging” role in the country cooperation.

#### Country Timeline Map

The results of country timeline map showed that the United States [22], Italy [23] and China [5] mainly focused on autoimmunity, and the attention had continued to be popular since 2004 till now. Norway [24], Poland [25] and Hungary [26] mainly studied SLE, and the focus was mainly from 2018 to 2021. France [27], Sweden [28], and Mexico paid more attention to B cell activating factor (BAFF), among which France paid more attention, and the time span was longer, from 2004 to now, especially from 2014 to 2021. Meanwhile, India, Japan and Canada were interested in reactive oxygen species, but the number of their publications decreased in the past two years (Fig. 3).

### Institution Distribution of Publications

#### Institution Co-occurrence Network

**Fig. 4:**
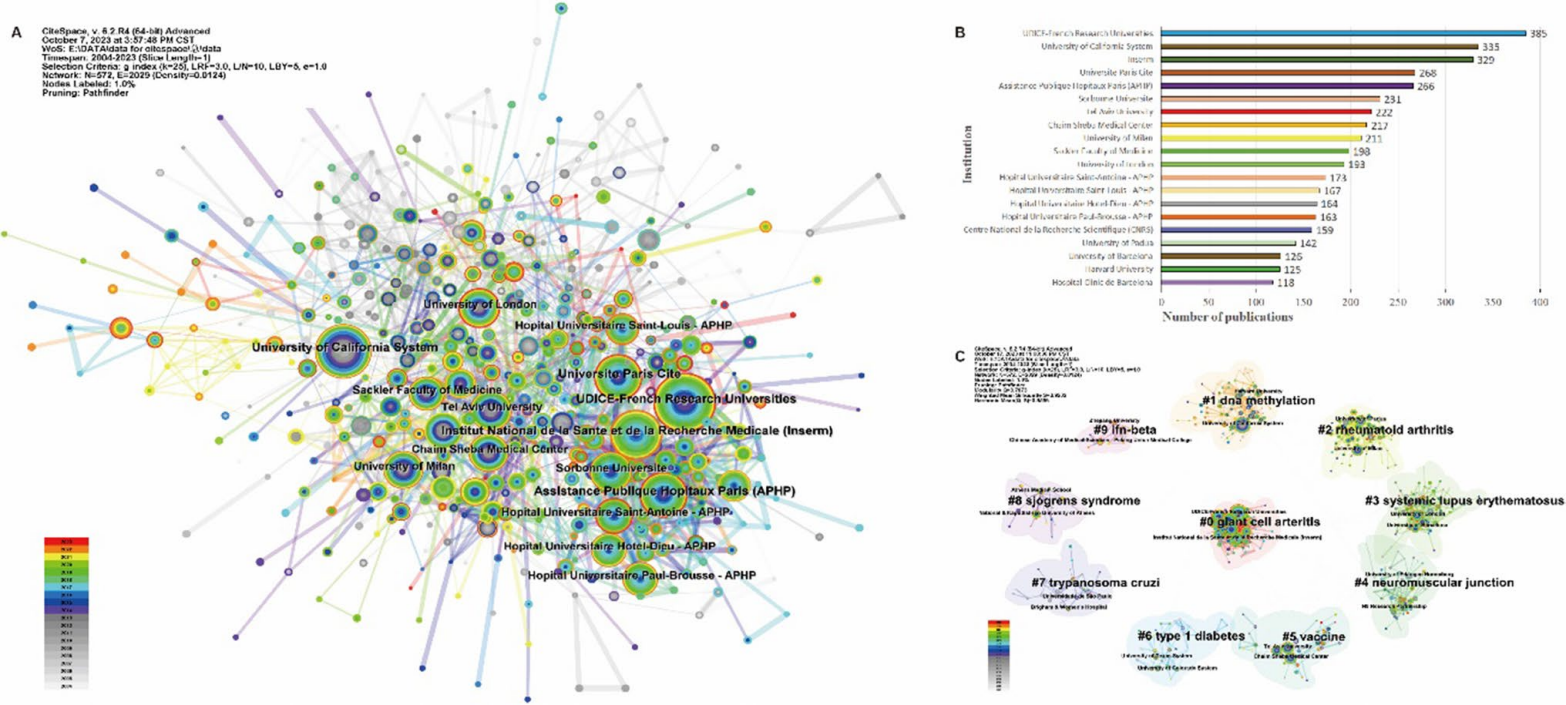
Analysis of the institution distribution of publications. *Note* A: the co-occurrence network of institutions; B: top 20 institutions by number of publications; C: the clustering map of institutions

The Citespace software integrates various aspects of information from literature, including co-occurrence patterns, citation relationships, keywords, and topics, to automatically cluster institutions. Using Citespace’s automatic clustering, 574 publishing institutions were identified and categorized into 10 groups (Fig. 4). Among these institutions, UDICE-French Research Universities, University of California System and Institut National de la Sante et de la Recherche Medicale (Inserm) ranked the top three in terms of publication numbers, belonging to France, the United States and France and with 385, 335 and 329 publications, respectively. Among the top 20 institutions in the number of publications, 8 were affiliated to France, 4 to the United States, 3 to Israel, 2 to Italy, 2 to Spain and 1 to the United Kingdom, which may be one of the reasons why the United States, Italy, and France lead in the number of publications (Fig. 4). However, China and Germany, which also ranked in the top 5% of the number of publications, had no institutions in the top 20, might be because the number of publications by each institution is relatively scattered and average.

#### Institution Clustering Map

By clustering the research directions of institutions, there were 10 categories: #0 “giant cell arteritis,” #1 “DNA methylation,” #2 “rheumatoid arthritis (RA),” #3 “systemic lupus erythematosus (SLE),” #4 “neuromuscular junction,” #5 “vaccine,” #6 “type 1 diabetes,” #7 “trypanosoma cruzi,” #8 “sjogrens syndrome (SS),” #9 “ifn-beta” (Fig. 4). Among them, the popularity of #0 “giant cell arteritis” was mainly contributed by UDICE-French Research Universities [29] with the largest number of publications and Inserm [30] with the third largest number of publications. The University of California System [31] with the second largest number of publications paid more attention to #1 “DNA methylation.”

### Author Distribution of Publications

#### Author Co-occurrence Network

**Fig. 5:**
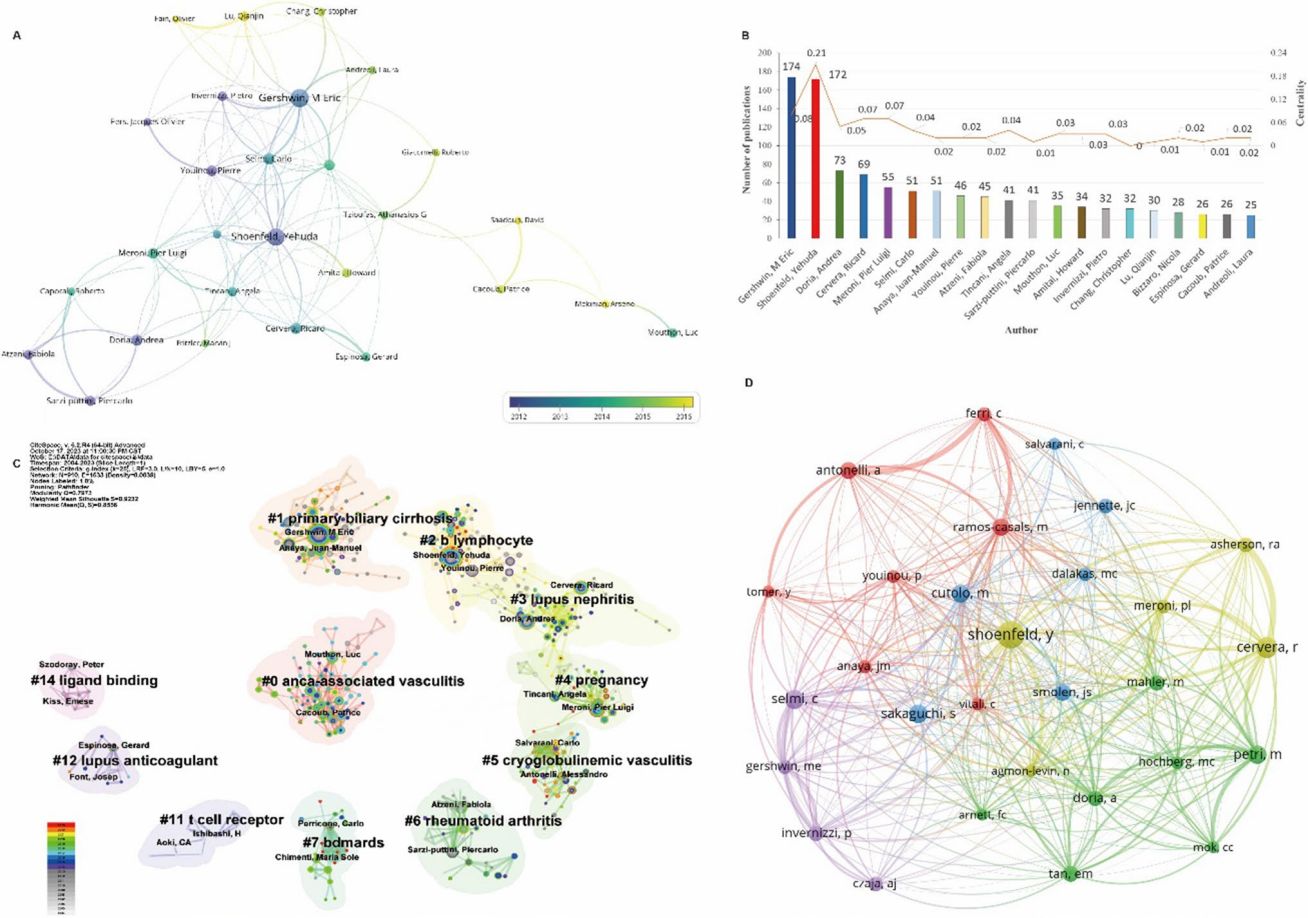
Analysis of the author distribution of publications. *Note* A: the co-occurrence network of authors; B: top 20 authors by number of publications and their centrality; C: the clustering map of authors; D: the co-cited network of authors

The number of scholarly publications of an author can represent the research activities and contributions in the field to some extent. The top three authors in the number of publications were Eric Gershwin, Yehuda Shoenfeld and Andrea Doria, who were affiliated with the University of California, Tel Aviv University and University of Padua, that were institutions ranking first, 8th and 18th in terms of the number of publications, respectively (Fig. 5). Eric Gershwin had published 174 papers, accounting for 51.94% of the total number of publications by the University of California System. Yehuda Shoenfeld, the author with the second largest number of publications, came from Israel, with 172 publications, accounting for 43.00% of the total number of publications in Israel. The author Andrea Doria, who ranked third in the number of publications, came from the University of Padua in Italy, with 73 publications, accounting for 57.94% of the total number of publications of the University of Padua. Among the top 10 authors in the number of publications, 5 authors were all from Italy, contributing to Italy ranking second in the number of publications.

#### Author Clustering Map

Author clustering results (Fig. 5) showed that many authors paid more attention to the topic of “ANCA-associated vasculitis,” among which Luc Mouthon [32] and Patrice Cacoub [33] had a large number of publications. Eric Gershwin [34] with the first number of publications and Juan-Manuel Anaya [35] with the seventh number of publications focused on “primary biliary cirrhosis,” and they had close cooperation. Shoenfeld Yehuda [36] and Pierre Youinou [37] were interested in the functional study of “B lymphocyte” and published many articles, ranking second and eighth, respectively. Andrea Doria [38] and Ricard Cervera [39], as the third and fourth authors in the number of publications, paid more attention to “lupus nephritis.”

#### Co-cited Author Analysis

Co-author analysis is a powerful method to describe the association between the authors, institutions and countries in a particular research field or a journal. The results of co-cited author analysis showed that the top five cited authors were Yehuda Shoenfeld, Ricard Cervera, Carlo Selmi, Shimon Sakaguchi and Michelle Petri, with cumulative citations reaching 520, 361, 319, 310 and 308 times, respectively (Fig. 5). Professor Yehuda Shoenfeld was the founder of the Zabludowicz Center for Autoimmune Diseases at the Sheba Medical Center. His clinical and scientific works focused on autoimmune and rheumatic diseases. He had published more than 1960 papers in many top journals, and these publications had been cited more than 55,000 times, some of which had become cornerstones in science and clinical practice. Professor Ricard Cervera, from Barcelona, was a key member of several Barcelona institutes for autoimmune diseases. His current major research interest included clinical and epidemiological aspects of systemic autoimmune diseases, particularly SLE and the antiphospholipid syndrome. Dr. Cervera had published more than 800 scientific papers, which had a great guiding role in the field of SLE research. Professor Carlo Selmi was leading the Rheumatology and Clinical Immunology Unit at Humanitas Research Hospital. Since 2009, he was the responsible of the Autoimmunity and Metabolic Laboratory, dedicated to the study of the possible mechanism linked to the progression of autoimmune diseases and chronic inflammatory diseases, including the clinical epidemiology and pathogenetic mechanisms of autoimmune liver disease, connective tissue disease (particularly systemic sclerosis), and psoriatic arthritis. He had coauthored over 400 publications with a D-index of 84 and citations nearly 20,000 times.

### Citation Analysis

#### The Highly Cited Publications

There were 33 highly cited papers in JDTA, including 26 reviews and 7 articles (Table 1). Of these papers, 22 were from Autoimmunity Reviews and 11 were from Journal of Autoimmunity. There were 16 articles related to COVID-19 (ranked 1, 2, 6, 9, 10, 15, 17, 18, 20, 23, 25, 26, 27, 28, 30, 31, respectively): 1 and 15 described the COVID-19 pandemic in detail, including the clinical manifestations, epidemiological characteristics, pathogenesis, control strategies; 2, 9, 10 explored the role of cytokines, especially interleukin-6, in the occurrence and development of COVID-19; 6, 17 investigated the possible therapeutic effects of tocilizumab and convalescent plasma on COVID-19, respectively; 18, 20, 23, 31 were reviews of the association between COVID-19 and autoimmunity, especially RA; 25, 26 described the characteristics of SARS-CoV-2 and its variants and their effects on the original antibodies; 27 was a meta-analysis and a review focusing on the protective effect of SARS-CoV-2 vaccines in patients with immune-mediated inflammatory diseases; 28 discussed the utility, importance, development prospects and vaccination strategies of SARS-CoV-2 vaccines; 30 comprehensively described the Post-COVID-19 syndrome, especially changes in autonomic nervous system function, and introduced some possible treatment measures. The above literatures have comprehensively studied and analyzed the etiology, symptoms, diagnosis, treatment, vaccine, complications, prognosis and other aspects of COVID-19.

**Table 1:** The highly cited papers

Rank	Article title	Authors	Journal title	Publication year	Times cited	Document type	DOI
1	The epidemiology and pathogenesis of coronavirus disease (COVID-19) outbreak	Rothan HA et al	Journal of autoimmunity	2020	2733	Review	10.1016/j.jaut.2020.102433
2	The role of cytokines including Interleukin-6 in COVID-19 induced pneumonia and macrophage activation syndrome-like disease	McGonagle D et al	Autoimmunity reviews	2020	1025	Review	10.1016/j.autrev.2020.102537
3	Th17 and regulatory T cell balance in autoimmune and inflammatory diseases	Noack M et al	Autoimmunity reviews	2014	621	Review	10.1016/j.autrev.2013.12.004
4	Vitamin D effects on musculoskeletal health, immunity, autoimmunity, cardiovascular disease, cancer, fertility, pregnancy, dementia and mortality-a review of recent evidence	Pludowski P et al	Autoimmunity reviews	2013	576	Review	10.1016/j.autrev.2013.02.004
5	Innate and adaptive immunity in inflammatory bowel disease	Geremia A et al	Autoimmunity reviews	2014	564	Review	10.1016/j.autrev.2013.06.004
6	Tocilizumab for the treatment of severe COVID-19 pneumonia with hyperinflammatory syndrome and acute respiratory failure: a single center study of 100 patients in Brescia, Italy	Toniati P et al	Autoimmunity reviews	2020	549	Review	10.1016/j.autrev.2020.102568
7	Hashimoto thyroiditis: clinical and diagnostic criteria	Caturegli P et al	Autoimmunity reviews	2014	488	Review	10.1016/j.autrev.2014.01.007
8	Autoimmune thyroid disorders	Antonelli A et al	Autoimmunity reviews	2015	486	Review	10.1016/j.autrev.2014.10.016
9	Can we use interleukin-6 (IL-6) blockade for coronavirus disease 2019 (COVID-19)-induced cytokine release syndrome (CRS)?	Liu BW et al	Journal of autoimmunity	2020	466	Review	10.1016/j.jaut.2020.102452
10	Should we stimulate or suppress immune responses in COVID-19? Cytokine and anti-cytokine interventions	Jamilloux Y et al	Autoimmunity reviews	2020	420	Review	10.1016/j.autrev.2020.102567
11	Zinc and its role in immunity and inflammation	Bonaventura P et al	Autoimmunity reviews	2015	419	Review	10.1016/j.autrev.2014.11.008
12	Obesity in autoimmune diseases: Not a passive bystander	Versini M et al	Autoimmunity reviews	2014	413	Review	10.1016/j.autrev.2014.07.001
13	Chemokine (C-X-C motif) ligand (CXCL)10 in autoimmune diseases	Antonelli A et al	Autoimmunity Reviews	2014	406	Review	10.1016/j.autrev.2013.10.010
14	The immunogenetics of Psoriasis: a comprehensive review	Harden JL et al	Journal of Autoimmunity	2015	364	Review	10.1016/j.jaut.2015.07.008
15	The deadly coronaviruses: the 2003 SARS pandemic and the 2020 novel coronavirus epidemic in China	Yang YS et al	Journal of Autoimmunity	2020	331	Review	10.1016/j.jaut.2020.102434
16	Neutrophil extracellular traps (NETs) in autoimmune diseases: a comprehensive review	Lee KH et al	Autoimmunity Reviews	2017	312	Review	10.1016/j.autrev.2017.09.012
17	Convalescent plasma in Covid-19: possible mechanisms of action	Rojas M et al	Autoimmunity Reviews	2020	294	Review	10.1016/j.autrev.2020.102554
18	Covid-19 and autoimmunity	Ehrenfeld M et al	Autoimmunity Reviews	2020	284	Review	10.1016/j.autrev.2020.102597
19	Systemic lupus erythematosus: diagnosis and clinical management	Fava A et al	Journal of Autoimmunity	2019	279	Article	10.1016/j.jaut.2018.11.001
20	COVID-19 infection and rheumatoid arthritis: faraway, so close!	Favalli EG et al	Autoimmunity Reviews	2020	265	Review	10.1016/j.autrev.2020.102523
21	Molecular mimicry and autoimmunity	Rojas M et al	Journal of Autoimmunity	2018	241	Article	10.1016/j.jaut.2018.10.012
22	The etiology of rheumatoid arthritis	Scherer HU et al	Journal of Autoimmunity	2020	237	Review	10.1016/j.jaut.2019.102400
23	The SARS-CoV-2 as an instrumental trigger of autoimmunity	Dotan A et al	Autoimmunity Reviews	2021	231	Article	10.1016/j.autrev.2021.102792
24	The 2022 outbreak and the pathobiology of the monkeypox virus	Kumar N et al	Journal of Autoimmunity	2022	161	Article	10.1016/j.jaut.2022.102855
25	Evolutionary analysis of the delta and delta plus variants of the SARS-CoV-2 viruses	Kannan S et al	Journal of Autoimmunity	2021	153	Article	10.1016/j.jaut.2021.102715
26	Omicron SARS-CoV-2 variant: unique features and their impact on pre-existing antibodies	Kannan S et al	Journal of Autoimmunity	2022	119	Article	10.1016/j.jaut.2021.102779
27	Response to SARS-CoV-2 vaccination in immune-mediated inflammatory diseases: systematic review and meta-analysis	Jena A et al	Autoimmunity Reviews	2022	102	Review	10.1016/j.autrev.2021.102927
28	Mitigating Covid-19 in the face of emerging virus variants, breakthrough infections and vaccine hesitancy	Haque A et al	Journal of autoimmunity	2022	63	Review	10.1016/j.jaut.2021.102792
29	The influence of cytokines on the complex pathology of ulcerative colitis	Nakase H et al	Autoimmunity reviews	2022	53	Review	10.1016/j.autrev.2021.103017
30	The autonomic aspects of the post-COVID19 syndrome	Dotan A et al	Autoimmunity reviews	2022	47	Review	10.1016/j.autrev.2022.103071
31	Autoantibodies and SARS-CoV2 infection: the spectrum from association to clinical implication: report of the 15th Dresden symposium on autoantibodies	Damoiseaux J et al	Autoimmunity reviews	2022	39	Review	10.1016/j.autrev.2021.103012
32	JAK inhibitors and autoimmune rheumatic diseases	Benucci M et al	Autoimmunity reviews	2023	10	Article	10.1016/j.autrev.2023.103276
33	Autoimmune pre-disease	Bieber K et al	Autoimmunity reviews	2023	8	Review	10.1016/j.autrev.2022.103236

The remaining 17 papers were all related to autoimmunity except for the 24th. 24 elaborated the 2022 outbreak and the pathobiology of the monkeypox virus. A total of 16 papers related to autoimmunity: 3, 13, 16, 21 discussed the role of Th17, regulatory T cell, chemokine ligand 10, neutrophil extracellular traps and molecular mimicry in the development of autoimmune diseases, respectively; 4, 11, 12 explored the effect of VitD, Zinc and obesity on body health, immunity and inflammation, respectively. 5, 7, 8, 14, 19, 22, 29, 32 gave a comprehensive description of various autoimmune diseases, including inflammatory bowel disease (5, 29), autoimmune thyroid disorders (7, 8), Psoriasis (14), SLE (19), and RA (22, 32).

#### The Top 20 Cited References

As shown in Table 2, the top 20 cited references could be divided into 6 categories: First, the reference ranked 1 formed unified, standardized and specific definitions of common vasculitis; Second, ranked 2, 5, 8, 16 references discussed the role of cell damage, epigenetics, protective T cell responses, regulatory T cells involved in the mechanisms of autoimmune diseases; Third, the references ranked 3, 12, 15, 18, 19 were the research of some specific autoimmune diseases, including SLE, systemic sclerosis, thyroid autoimmunity, primary biliary cirrhosis and SS; Fourth, the references ranked 4, 7, 13, 14 introduced the achievements of four outstanding immunologists, Susumu Ikehara; Ian Reay Mackay, Noel Rose, and Haralampos M. Moutsopoulo; Fifth, the references ranked 6, 10, 11 described the geoepidemiology and its association with autoimmunity; Sixth, the references ranked 9, 17, 20 explored the relationship between gender, environment, pregnancy and autoimmunity, respectively.

**Table 2:** The top 20 cited references of publications in JDTA

Rank	Reference title	Authors	Journal title	Publication year	Total citations	Document type
1	2012 revised international Chapel Hill consensus conference nomenclature of vasculitides	Jennette JC et al	Arthritis and Rheumatism	2013	58	10.1002/art.37715
2	Cell damage and autoimmunity: a critical appraisal	Mackay IR et al	Journal of autoimmunity	2008	44	10.1016/j.jaut.2007.11.009
3	Development of autoantibodies before the clinical onset of systemic lupus erythematosus	Arbuckle MR et al	New England Journal of Medicine	2003	41	10.1056/NEJMoa021933
4	Bone marrow transplantation, refractory autoimmunity and the contributions of Susumu Ikehara	Gershwin ME et al	Journal of Autoimmunity	2008	40	10.1016/j.jaut.2007.12.006
5	Epigenetics and autoimmunity	Brooks WH et al	Journal of Autoimmunity	2010	38	10.1016/j.jaut.2009.12.006
6	The autoimmunologist: geoepidemiology, a new center of gravity, and prime time for autoimmunity	Shoenfeld Y et al	Journal of Autoimmunity	2008	34	10.1016/j.jaut.2008.08.004
7	A tribute to an outstanding immunologist—Ian Reay Mackay	Whittingham S et al	Journal of Autoimmunity	2008	32	10.1016/j.jaut.2008.04.004
8	Balancing autoaggressive and protective T cell responses	Abbas AK et al	Journal of Autoimmunity	2007	32	10.1016/j.jaut.2007.02.002
9	Gender as risk factor for autoimmune diseases	Gleicher N et al	Journal of Autoimmunity	2007	31	10.1016/j.jaut.2006.12.004
10	Geoepidemiology and autoimmunity	Youinou P et al	Journal of Autoimmunity	2010	31	10.1016/j.jaut.2009.12.005
11	Defining and analyzing geoepidemiology and human autoimmunity	Shapira Y et al	Journal of Autoimmunity	2010	29	10.1016/j.jaut.2009.11.018
12	Systemic sclerosis	Denton CP et al	Lancet	2017	28	10.1016/S0140-6736(17)30,933-9
13	Navigating the passage between Charybdis and Scylla: recognizing the achievements of Noel Rose	Ansari AA et al	Journal of Autoimmunity	2009	28	10.1016/j.jaut.2009.07.007
14	Haralampos M. Moutsopoulos: a lifetime in autoimmunity	Youinou P et al	Journal of Autoimmunity	2010	26	10.1016/j.jaut.2010.06.003
15	The HLA gene complex in thyroid autoimmunity: from epidemiology to etiology	Jacobson EM et al	Journal of Autoimmunity	2008	25	10.1016/j.jaut.2007.11.010
16	Regulatory T cells and immune tolerance	Sakaguchi S et al	Cell	2008	25	10.1016/j.cell.2008.05.009
17	Mechanisms of environmental influence on human autoimmunity: a national institute of environmental health sciences expert panel workshop	Selmi C et al	Journal of Autoimmunity	2012	25	10.1016/j.jaut.2012.05.007
18	IL-2 receptor alpha deficiency and features of primary biliary cirrhosis	Aoki CA et al	Journal of Autoimmunity	2006	25	10.1016/j.jaut.2006.04.005
19	Pathogenesis of Sjögren’s syndrome: what we know and what we should learn	Tzioufas AG et al	Journal of Autoimmunity	2012	24	10.1016/j.jaut.2012.01.002
20	The implications of autoimmunity and pregnancy	Borchers AT et al	Journal of Autoimmunity	2010	24	10.1016/j.jaut.2009.11.015

### Keyword Analysis

#### Keywords Co-occurrence Network

**Fig. 6:**
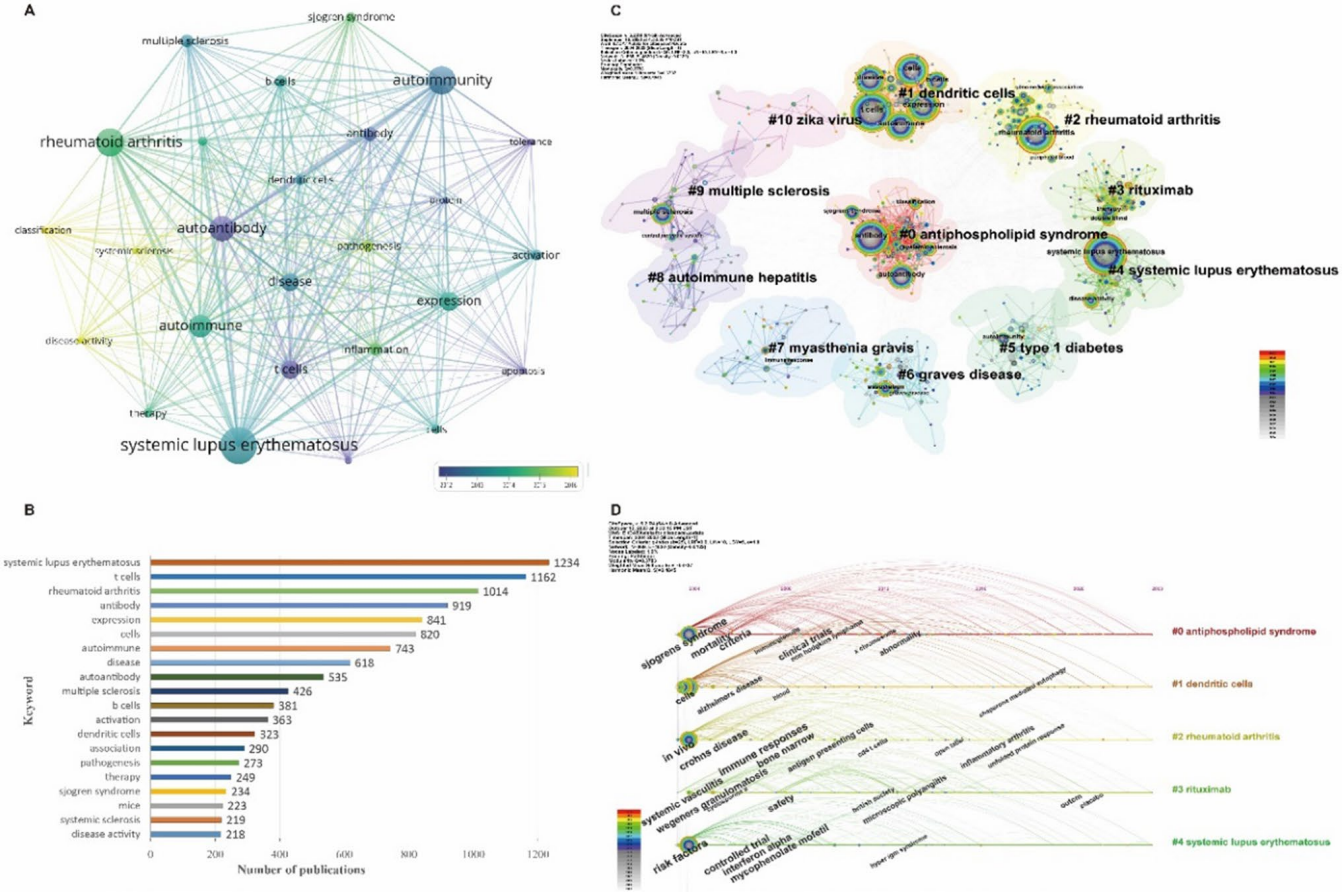
Analysis of the keyword distribution of publications. *Note* A: the co-occurrence network of keywords; B: top 20 keywords by number of publications; C: the clustering map of keywords; D: the timeline map of keywords

All key words were extracted from all the 5,170 publications, and those with the same meaning were merged. Finally, there were 303 keywords that appeared more than 10 times, among which the co-occurrence network of the top 25 keywords was shown in Fig. 6. In this mapping, the size of the node was proportional to the number of occurrences of the keyword. According to the corresponding year of occurrence of these keywords, different colors mark different years. It can be seen that “systemic lupus erythematosus,” “t cells, rheumatoid arthritis,” “antibody,” “expression,” “cells,” “autoimmune,” “disease,” “autoantibody” and “multiple sclerosis” received the most attention, with the number of publications of 1,234, 1,162, 1,014, 919, 841, 820, 743, 618, 535, 426, respectively (Fig. 6).

#### Keywords Clustering Map and Timeline Map

As shown in Fig. 6, the keywords were clustered into 11 categories, namely #0 antiphospholipid syndrome, including antibody, autoantibody, SS, systemic sclerosis, classification and so on; #1 dendritic cells, including t cells, expression, cells, autoimmune, disease, b cells, and so on; #2 RA, including RA, peripheral blood, genome wide association and so on; #3 rituximab, including therapy, double blind and so on; #4 SLE, including SLE, disease activity and so on; #5 type 1 diabetes; #6 graves disease, including graves disease, association and so on; #7 myasthenia gravis, #8 autoimmune hepatitis, #9 multiple sclerosis, including multiple sclerosis, central nervous system and so on; and #10 zika virus.

## Keywords with Strongest Citation Bursts

**Fig. 7:**
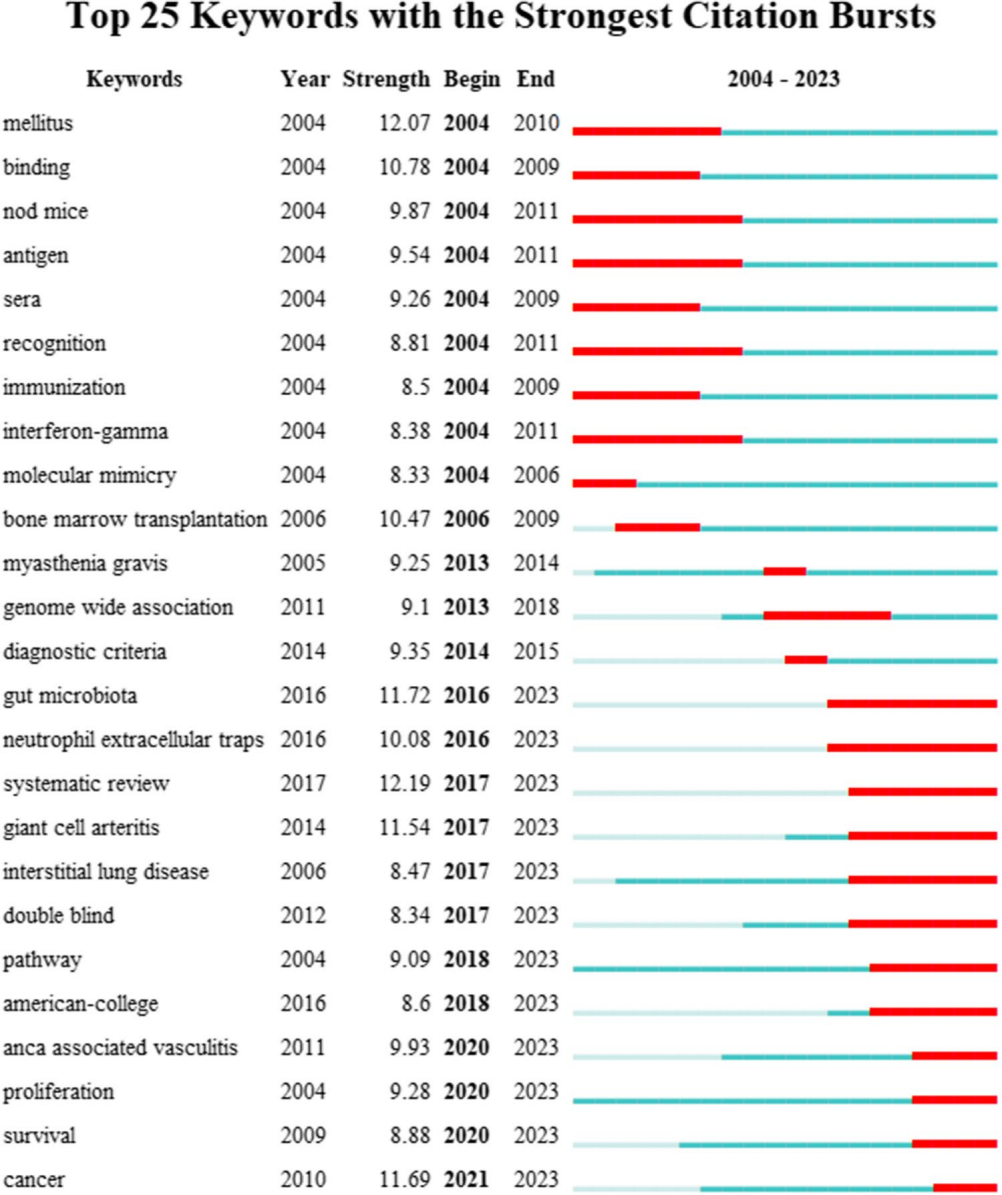
The top 25 keywords with the strongest citation bursts

Burst analysis was used to capture keywords with a sharp increase in popularity during a certain period of time. Figure 7 showed the top 25 keywords with the strongest burst. As shown in Fig. 7, the keyword with the strongest burst was "genome wide association (GWAS)", with a strength of 14.13, and the burst lasted from 2013 to 2018. This might be due to that with the use of GWAS methods in various research fields, related publications of GWAS in the field of autoimmune diseases also sprung up. Meanwhile, the burst strength of “systematic review” and “mellitus” was 13.39 and 13.38, ranking second and third, respectively. The burst of “systematic review” occurred from 2017 to now, suggesting that systematic review, as the most systematic and rapid means to understand a certain research field, was still concerned and recognized by researchers. However, the burst time of “mellitus” was earlier, between 2004 and 2008, when attention to diabetes, especially type 1 diabetes mellitus, increased dramatically.

## Discussion

This bibliometric analysis study provides a first comprehensive overview of publications in JDTAs, including three journals, Autoimmunity Review Journal of Autoimmunity and Autoimmunity, over the last two decades, from 2004 to 2023. Since 2004, the annual number of JDTA publications had been basically stable, fluctuating around 300, suggesting that the development of these three journals was stable and mature, and they strictly control the quantity and quality of publications.

Globally, the United States and Italy led the way in terms of the number of publications, followed by France and China. According to the results of national analysis, United States and Italy began to research in the related field and published a large number of publications since 2004, especially the United States, of which publications from 2004 to 2013 accounted for a large proportion of its all publications. France began to have a large number of publications in 2015, focusing on 2015–2019. At the same time, China began to publish a large number of studies in 2017, and the number of publications has also maintained at a high level since then. In addition, the results of country clustering based on keywords showed that the France and United States had a good cooperative relationship with other countries. For international cooperation, the developed countries represented by the United States cooperate more closely, but the cooperation was localized, reflecting that there was no unified model of autoimmunity among countries. Therefore, we believe that cooperation between countries in the same research field is very likely to promote each other's research progress, while cooperation between countries with different focuses can effectively fill research gaps in their respective fields.

In terms of research institutions, UDICE-French Research Universities published the most articles, and subsequently, the number of publications decreased slowly with the ranking, with no large gradient. There was extensive cooperation among research institutions, especially several French institutions in the top 20, which were closely connected and cooperated well. However, the cooperation among other institutions was weak and still needed to be strengthened. None of top 20 institutions were from China and Germany, which ranked fourth and fifth in the number of publications, respectively, which may be due to the relatively dispersed and average number of publications by institutions in these two countries. Hence, for institutions in China and Germany, strengthening cooperation between them, focusing on and assisting leading research institutions, helps enhance the influence of respective institutions, accelerates the research process, improves research quality, and facilitates wider sharing and utilization of research outcomes among different institutions.

Eric Gershwin and Yehuda Shoenfeld stood out among the many authors because of their excellent academic reputation and dominance in the field of autoimmunity. Eric Gershwin mainly focused on immunology, primary biliary cirrhosis and autoimmunity. The concepts of his autoimmunity study were interwoven with issues in autoimmune disease, molecular mimicry, immune tolerance and genetic predisposition. Eric Gershwin worked closely with authors Carlo Selmi and Juan-Manuel Anaya, who ranked 6th and 7th in the number of publications, respectively. They had collaborated on several publications focusing on autoimmune diseases, especially primary biliary cirrhosis. Yehuda Shoenfeld, as the most cited author of JDTA, also has a great influence in the field. Yehuda Shoenfeld worked mostly in the field of autoantibody, limiting it down to topics relating to adjuvant and, in certain cases, autoimmune/inflammatory syndrome induced by adjuvants. His research integrated issues of SLE and Anti-SSA/Ro autoantibodies in his study of autoantibody. However, the author analysis also suggested that the density of links between authors was not enough and still needed to be strengthened. In terms of authors, notable research achievements in the field of primary biliary cholangitis have been made by scholars Eric Gershwin, Carlo Selmi, Juan-Manuel Anaya et al. Collaborations among these esteemed authors have the potential to greatly drive research advancements in primary biliary cholangitis.

Collaborative efforts among these esteemed authors hold immense promise for propelling research progress in primary biliary cholangitis.

The results of keyword analysis showed that JDTA publications mainly studied a variety of autoimmune diseases, especially SLE and RA. At the same time, JDTA publications also paid special attention to the research of cell function, autoantibody expression, animal experiments, disease activity, pathogenesis and treatment. Based on the information from emerging keywords, we cannot only gain insights into past hotspots in research fields but also can preliminarily predict future keywords that might become popular. Firstly, terms related to health and biomedical fields, such as “gut microbiota” and “immune system,” due to their recent high citation intensity, may continue to be popular. Additionally, with the development of technology and research, new research directions and fields may emerge, bringing forth new popular keywords. For example, in fields like genomics, precision medicine, and the integration of artificial intelligence with medicine, new attention-grabbing keywords may arise. This feature of Citespace reminds us that it is not just a tool for bibliometrics; with the development of the big data era, AI technology also holds significant importance in analyzing research hotspots and predicting trends, deserving further exploration and consideration.

Some limitations still exist in our studies. Firstly, although the WOSCC database had the most complete coverage of articles, some papers from different databases cannot be retrieved. Secondly, given our study focus on bibliometric analysis of JDTA, and the number of eligible journals is relatively limited; we opted not to compare these three target journals separately to ensure a comprehensive overview of the field. Thirdly, we only analyzed articles and reviews and excluded meeting, letter, case report, editorial material, abstract, retractions. These may lead to incomplete data collection and different results.

## Conclusion

This bibliometric analysis study provides a comprehensive overview of JDTA over the last two decades. The number of JDTA publications was basically stable, fluctuating around 300 yearly. Globally, the United States and Italy were in the leading positions in terms of the number of publications. Among the Research institutions, UDICE-French Research Universities had the greatest impact on publications. Eric Gershwin and Yehuda Shoenfeld contributed greatly to the publications of POAT. Different countries, institutions and authors needed to strengthen cooperation and communication. The publications of JDTA mainly studied a variety of autoimmune diseases, especially SLE and RA. JDTA focused on cell function, autoantibody expression, animal experiments, and disease mechanisms. They represented the current research and the development trend of future researches in the field of autoimmunity.

## Announcement of conflict of interest

All authors declare that no potential conflict of interest exists, consisting any financial or commercial ties.

## Data Availability

The datasets utilized in the current study are contained in the article/Supplementary material.
